# SKY analysis revealed recurrent numerical and structural chromosome changes in BDII rat endometrial carcinomas

**DOI:** 10.1186/1475-2867-11-20

**Published:** 2011-06-27

**Authors:** Eva Falck, Carola Hedberg, Karin Klinga-Levan, Afrouz Behboudi

**Affiliations:** 1 Systems Biology Research Centre, School of Life Sciences, University of Skövde, SE-54128 Skövde, Sweden; 2 Department of Medical and Clinical Genetics, Institute of Biomedicine, University of Gothenburg, SE-40530 Gothenburg, Sweden

**Keywords:** SKY, BDII rat, endometrial carcinoma

## Abstract

**Background:**

Genomic alterations are common features of cancer cells, and some of these changes are proven to be neoplastic-specific. Such alterations may serve as valuable tools for diagnosis and classification of tumors, prediction of clinical outcome, disease monitoring, and choice of therapy as well as for providing clues to the location of crucial cancer-related genes.

Endometrial carcinoma (EC) is the most frequently diagnosed malignancy of the female genital tract, ranking fourth among all invasive tumors affecting women. Cytogenetic studies of human ECs have not produced very conclusive data, since many of these studies are based on karyotyping of limited number of cases and no really specific karyotypic changes have yet been identified. As the majority of the genes are conserved among mammals, the use of inbred animal model systems may serve as a tool for identification of underlying genes and pathways involved in tumorigenesis in humans. In the present work we used spectral karyotyping (SKY) to identify cancer-related aberrations in a well-characterized experimental model for spontaneous endometrial carcinoma in the BDII rat tumor model.

**Results:**

Analysis of 21 experimental ECs revealed specific nonrandom numerical and structural chromosomal changes. The most recurrent numerical alterations were gains in rat chromosome 4 (RNO4) and losses in RNO15. The most commonly structural changes were mainly in form of chromosomal translocations and were detected in RNO3, RNO6, RNO10, RNO11, RNO12, and RNO20. Unbalanced chromosomal translocations involving RNO3p was the most commonly observed structural changes in this material followed by RNO11p and RNO10 translocations.

**Conclusion:**

The non-random nature of these events, as documented by their high frequencies of incidence, is suggesting for dynamic selection of these changes during experimental EC tumorigenesis and therefore for their potential contribution into development of this malignancy. Comparative molecular analysis of the identified genetic changes in this tumor model with those reported in the human ECs may provide new insights into underlying genetic changes involved in EC development and tumorigenesis.

## Introduction

The most frequently diagnosed malignancy of the female genital tract is cancer of the endometrium. Endometrial carcinoma (EC) is the predominant sub type, ranking fourth among all invasive tumors that affect women. Approximately 85% of the patients diagnosed with this malignancy are over 50 years of age [1].

As most other cancer types, EC is a complex genetic disease as its development is influenced by multiple genetic alterations [2-5]. Cytogenetic studies of ECs have shown that most tumors have hyperdiploid karyotypes with relatively minor chromosomal aberrations [6]. The reported cytogenetic data are not conclusive, since they are based on the karyotyping of limited number of cases [6], and no really specific karyotypic changes have yet been detected. In general, genetic studies of complex diseases in human is proven to be difficult due to heterogeneity of the human population with respect to genetic background and diversity of the influencing environmental factors [7-9]. As the majority of the genes are conserved among mammals, the use of inbred animal model systems may serve as a powerful tool for identification of underlying genes and pathways in human disease phenotypes. There are many animal models available for studies of human disorders, among which a number of inbred rat model strains provide unique models for the analysis of cancer [10,11]. Of these, four develop EC spontaneously, of which females from the BDII/Han strain (hereafter BDII) is prone to develop tumors with the highest incidence (more than 90% among the virgin females) [12,13]. EC development in BDII rats has similarities in pathogenesis, histopathological and molecular properties to human EC, and thus the inbred BDII strain represents a unique model for analysis of EC tumorigenesis [14]. This tumor model has been genetically well characterized [3,4,15,16], but there still is much important genetic information to be fully understood [13].

Genomic alterations are common features of cancerous cells, which may appear as chromosomal aberrations, including numerical and structural changes [17]. In cytogenetic studies of neoplasms it is shown that a large fraction of chromosomal abnormalities in many cancer types are neoplastic-specific. Such findings might thus serve as valuable tools for diagnosis and classification of tumors, prediction of clinical outcome, disease monitoring, and the choice of therapy [18]. They additionally may provide clues to locations of crucial cancer-related genes involved in tumorigenesis and tumor progression pathways. Detailed analysis of these genes may offer valuable tools for early diagnosis and prognosis of cancer as well as for the drug discovery. In this regard, genomic approaches have proven to be effective in detecting chromosomal alterations pinpointing candidate genes that are involved in cancer development [17].

Spectral karyotyping (SKY) is a method used to detect aberrations and rearrangements through direct examination of metaphases and chromosomes. In SKY analysis, the chromosomes are labeled with their specific different dyes and thus different forms of chromosomal alterations are easily detected [19]. The SKY technique is very useful in clinical cytogenetics, in particular in the analysis of tumor cells, where multiple and complex chromosome aberrations are common [20,21].

Here, we report results from detailed cytogenetic analysis of a set of 21 BDII rat endometrial adenocarcinoma primary cell cultures using SKY technique. We found specific nonrandom chromosomal changes in his model with potential contribution to endometrial carcinogenesis.

## Materials and methods

### Tumor material

EC Susceptible BDII females (with incidence of more than 90%) were crossed to EC resistant SPRD-Cu3/Han and BN/Han males (hereafter SPRD and BN, with incidences of less than 10%). F1 progenies were backcrossed to the female rats of the susceptible parental strains (BDII) to produce backcrosses (N1), or intercrossed in brother-sister mating to produce F2 progeny. Spontaneously arising tumors developed in a proportion of F1, F2 and N1 progeny. All tumors were characterized histopathologically and the majority were classified as EC. The RUT (Rat Uterine Tumors) specimens represent ECs developed in the F1 and F2 progenies and NUT (N1 Uterine Tumor) specimens represent ECs developed in the backcross (N1) progeny. Small pieces of fresh tumor tissue were used to set up primary cell cultures [22]. Twenty-one of these primary tumor cell cultures were used in the present study (Table 1), 10 derived from crosses with the SPRD background and 11 with the BN background. A rat embryo fibroblasts (REF) cell culture was used as normal control [23]. All animal experiments was approved by the local ethical committee (Institute of Laboratory Animal Science and Central Animal Facility, Hannover Medical School, Germany).

**Table 1:** Twenty-one primary tumors cell lines derived from ECs in F1, F2 and N1 progeny after crosses between EC susceptible BDII females and EC non-susceptible SPRD and BN males.

Tumor	Background (cross)	Ploidy level				Total metaphases analyzed
			
		Diploidy	Triploidy	Tetraploidy	Others	
NUT3	SPRD (N1)	16	2	5		23
NUT7	SPRD (N1)	6	4	13	1	24
NUT12	SPRD (N1)	2	22		1	25
NUT29	SPRD (N1)	14	4	3		21
NUT39	SPRD (N1)		10	9	1	20
NUT42	SPRD (N1)	4	0	1	1	6
NUT47	SPRD (N1)	19	3	2		24
NUT84	SPRD (N1)	26				26
RUT2	SPRD (F1)	26				26
RUT6	SPRD (F2)	1	28			29
RUT13	SPRD (F2)	6	18			24
NUT6	BN (N1)	5	18			23
NUT50	BN (N1)	6	23		2	31
NUT52	BN (N1)	9	7	7		23
NUT97	BN (N1)	10	15			25
NUT98	BN (N1)	14				14
NUT100	BN (N1)	7	17			24
NUT127	BN (N1)	3	9	11		23
NUT128	BN (N1)	3	23			26
RUT7	BN (F1)	23		1	2	26
RUT25	BN (F2)	13	11	3		27

Background: genetic background of the animals that developed tumors (cross of BDII females to SPRD or BN males); Progeny: F1 - first generation intercross offspring; F2 - second generation intercross offspring; N1 - first back-cross generation offspring; Ploidy level: number of methaphases that showed diploid, triploid, tetraploid or other (near haploid, pentaploid and hexaploid) karyotype.

### Chromosome preparations

Cells were treated with Colcemid (0.05 ug/ml, Life Technologies, Grand Island, NY), harvested after 20 min by mitotic shake-off and pelletized by centrifugation. The pellet was re-suspended in 0.075 M KCl and left at room temperature for 15 min. Subsequently, fixation was carried out with methanol-acetic acid fixative series [24]. The chromosome spreads were air-dried and stored at room temperature for 5-6 days prior to the SKY experiments.

### Hybridization

Slides were pretreated with pepsin to minimize the non-specific binding and to reduce background fluorescence. The slides were then washed in a PBS and MgCl_2 _solution to stop the pepsin digestion and incubated in a solution of 1% formaldehyde in 1 × PBS/MgCl_2 _for 10 minutes to strengthen the chromosomal structure. The rat SKY probe (Applied Spectral Imaging, Israel, ASI) was denatured at 80°C for 7 minutes and then incubated at 37°C for 60 minutes. Metaphase slides were denatured in 70% formamide at 75°C for 2-3 minutes, 5 μl of the denatured probe was added to the denatured metaphase chromosomes and the hybridization was carried out for 48 hr at 37°C in a humidity chamber.

### Detection and image analysis

Following the hybridization step, excess of the probe was washed from the slides. The hybridized probes were then stained using anti-digoxin and Cy5 Strepavidin staining followed by a Cy5.5 sheep anti mouse antibody treatment. The chromosomes were counterstained with 4,6-diamino-2-phenylindole (DAPI) in an anti-fade solution (ASI). Imaging of the signals was carried out using the SpectraCube system mounted on a Zeiss Axioskop 2 Mot Plus Imaging microscope. The images were analyzed using the HiSKY^®^multispecies software (ASI).

## Results

**Figure 1:**
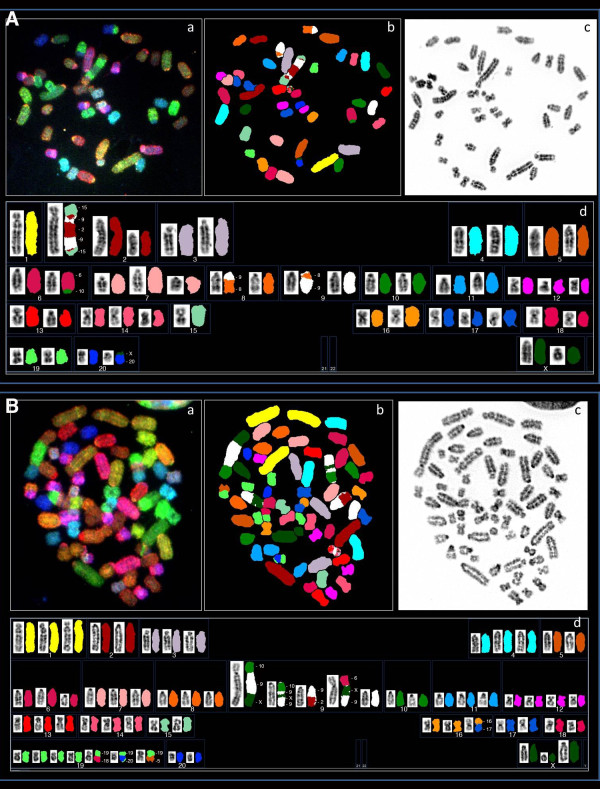
**Examples of depicted SKY analysis results for tumor samples**: A) NUT3, B) NUT128. **a**. RGB image, **b**. pseudo-colored image, **c**. inverted DAPI image (G-band), **d**. Complete SKY compared to G-banded karyotype.

In average 24 metaphases per tumor samples were analyzed, except for two tumors for which only 6 (NUT 42) and 14 (NUT98) analyzable metaphases were available (Table 1). The REF cell line displayed a normal diploid karyotype in all of the 25 metaphases analyzed. All tumor samples, but two (RUT2 and NUT84), showed a mixed population of clones with different ploidy grades (Table 1). The majority of tumors displayed a complex pattern of numerical and structural aberrations (Figure 1). Using the International System for Human Cytogenetic Nomenclature (ISCN 1995) and literature on nomenclature for G-bands in rat chromosome [25,26], we determined the most common cytogenetic changers among the tumors (Table 2).

**Table 2:** The most commonly structural aberrations in the tumors (identified in 75% or more of the metaphases analyzed).

Tumor	Range of chr. no. (% of metaphases in each group)	No. of metaphases	Recurrent chromosomal changes
NUT3	38-51 (70%), 61-66 (8%), 82-87 (22%)	23	t(2;9;15), t(6;10), t(8;9), der(X)
NUT6	40-51 (22%), 58-71 (78%)	23	t(1;20), der(1), der(2), t(3;6;9), t(9;6;3;6;9), t(9;11), der(10), t(7;12), t(12;17)
NUT7	39-45 (25%), 56-73 (17%), 74-90 (54%), 138 (4%)	24	t(1;X;5;4), t(5;14), t(10;12), t(5;15), t(X;10)
NUT12	38-45 (8%), 56-64 (88%) 114 (4%)	25	t(1;3), t(1;9), t(3;4), t(4;12), t(X;6), t(8;11), der(9), t(10;15), t(11;18), t(18;19)
NUT29	38-49 (67%), 68-73 (19%), 80-86 (14%)	21	t(9;10), many other different translocations
NUT39	57-73 (50%), 74-81 (45%), 141 (5%)	20	t(3;8), t(3;5), HSR on chr. 4, t(6;12), der(7), t(12;17),
NUT42	26 (17%), 37-43 (67%), 79 (17%)	6	numerical aberrations only
NUT47	40-52 (79%), 53 (13%), 90-93 (8%)	24	der(X), numerical aberrations only
NUT50	21-30 (6%), 35-45 (19%), 55-68 (74%)	31	t(3;8), t(9:10), t(3;10), t(8;17), der(X)
NUT52	40-45 (39%), 55-70 (30%), 74-80 (30%)	23	t(1;12), t(6;12), HSR on chr. 6, t(8;8), t(10;20), t(10;16)
NUT84	37-50 (100%)	26	t(2;6), t(2;6;3), t(2;6), t(5;6), t(6;16), t(5;8), der(10)
NUT97	48-52 (40%), 56-63 (60%)	25	del(3), t(3;6), t(7;18), t(2;9)
NUT98	35-49 (100%)	14	t(2;9), der(3), t(3;6), der(18), der(X)
NUT100	36-50 (29%), 61-66 (71%)	24	t(1;13/14), t(2;3), t(3;7;4), t(4;18), der(5), t(6;17), der(9), t(5;10), der(10), t(11;18)
NUT127	46-48 (13%), 60-73 (39%), 74-78 (48%)	23	t(5;13), t(8;10), t(1;20)
NUT128	40-52 (13%), 53-72 (88%)	26	der(2), der(6), t(2;9), t(X;9;10), t(6;X;9), t(12;13/14), t(16;17), t(5;19), t(12;19), t(18;19), t(19;20)
RUT2	34-52 (100%)	26	t(3;17), t(5;17), t(6;15), t(10;18), t(10;16), t(5;17)
RUT6	47 (3%), 54-71 (97%),	29	t(2;12), t(3;4), t(6;20), t(7;15), der(10), t(1;16), t(15;20)
RUT7	22-27 (8%), 32-45 (88%), 88 (4%)	26	t(3;8), t(5;1;4), der(4), t(1;5), t(2;8), der(10), 17-not present
RUT13	39-52 (25%), 53-62 (75%)	24	t(3;4;15), t(3;4), t(1;4), t(4;11), t(6;11), t(6;12), t(10;13/14), t(10;15), t(X;18)
RUT25	41-52 (48%), 53-68 (41%), 79-87 (11%)	27	t(3;11), t(1;10), t(17;19), der(X)

t: translocation; der: derivative; HSR: homogenously staining regions.

## Discussion

Chromosomal instability (CIN) is a common feature of most human cancers. CIN may result in imbalances in the chromosome numbers (aneuploidy) and/or enhanced rate of structural aberrations (translocation, inversion, deletion, insertion, etc). These changes may be important mechanisms of activating or inactivating of oncogenes and tumor suppressor genes, respectively. A crucial question of cancer etiology is then whether CIN is an early event and thus a driving force of tumorigenesis [27]. In general, detailed analysis of CIN in tumor cells is hampered by limitations in conventional banding techniques as well as by the complex nature of cancer-related chromosome aberrations in tumor cells. SKY is a molecular cytogenetic technique by which many forms of multiple and complex aberrations can easily be characterized. SKY has made it possible to detect the so-called hidden structural alterations, such as translocations in regions with similar banding patterns that could have been left otherwise undetected by the classical cytogenetic methods. In cancer cells, next after numerical chromosomal changes and translocations, formation of unidentifiable marker chromosomes of multiple chromosomal origins is common [28]. Identification of origins of such marker chromosomes has become easier by using SKY.

Cytogenetic analyses of human ECs have shown these tumors to mostly exhibit simple karyotypic abnormalities with few numerical and/or structural chromosomal rearrangements [29-31]. Despite this relative karyotypic simplicity, chromosomal aberrations with potential contribution to EC development have only been partially studied. In the present work, we used a powerful experimental model for spontaneous endometrial carcinogenesis to explore rates and features of chromosomal instability in EC. Twenty-one rat EC primary tumor cell cultures derived from solid tumors developed in the female progeny from crosses between EC susceptible BDII female rats and EC non-susceptible BN and SPRD male rats were subjected to detailed cytogenetic analysis using SKY. The majority of tumors displayed a complex pattern of numerical and structural aberrations (Table 2, Figure 1). To examine whether certain chromosomes were more frequently involved in aberrations as well as to identify the most recurrent changes, we calculated the total number of numerical and structural aberrations per chromosome in the tumor material (Tables 3 and 4).

**Table 3:** Analysis of numerical chromosome changes in 21 experimental EC tumors.

Chromosome	Observed	**Obs./Exp**.	% of gain (+) or loss (-)
1	1317	1.005	+0.46
2	1347	1.027	+2.75
3	1331	1.015	+1.53
4	1617	1.233	+23.34
5	1173	0.895	-10.53
6	1478	1.127	+12.74
7	1253	0.956	-4.42
8	1246	0.950	-4.96
9	1344	1.025	+2.52
10	1260	0.961	-3.89
11	1180	0.900	-9.99
12	1483	1.131	+13.12
13	1142	0.871	-12.89
14	1207	0.921	-7.93
15	1125	0.858	-14.19
16	1491	1.137	+13.73
17	1296	0.989	-1.14
18	1156	0.882	-11.82
19	1427	1.088	+8.85
20	1164	0.888	-11.21
X	1307	0.997	-0.31

The expected number of each chromosome in the tumor panel was calculated as 1311. The most recurrently gained and lost chromosomes are marked in gray and black, respectively.

**Table 4:** Numerical and structural aberrations detected in each chromosome in the tumor panel.

**Chr**.	Size (Mb)	No. of chromosome	Structural changes			
			
			Deletion	Translocation	Amplification	Total
1	267.9	1317	55	132		187
2	258.2	1347	33	184	9	226
3	171.1	1331	49	438		487
4	187.1	1617	94	265	30	389
5	173.1	1173	82	242	12	336
6	147.6	1478	68	426	45	539
7	143	1253	73	49	5	127
8	129	1246	19	188	3	210
9	113.4	1344	56	112	1	169
10	110.7	1260	192	219	1	412
11	87.8	1180	24	191	16	231
12	46.8	1483	13	179	5	197
13	111.2	1142	2	44		46
14	112.2	1207	11	41		52
15	109.8	1125	3	155	1	159
16	90.2	1491	15	116	3	134
17	97.3	1296	8	168	6	182
18	87.3	1156	17	117	4	138
19	59.2	1427	10	77	1	88
20	55.3	1164	1	145	3	149
X	160.7	1307	116	82	39	237

"Amplification" represents both the observed HSR and double minutes in metaphases.

**Figure 2:**
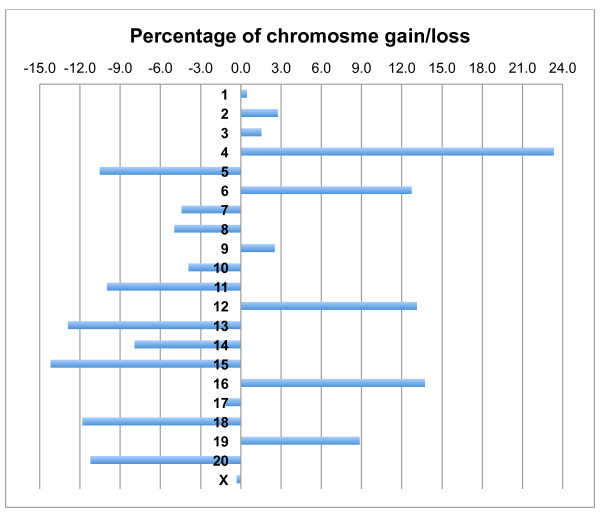
**Numerical chromosome changes in 21 experimental EC tumors as identified by SKY analysis**. As shown, chromosomal gains are less common, but more profound compared to chromosomal losses.

To identify non-random numerical chromosome aberrations, we calculated the expected and observed numbers of chromosomes in all metaphases analyzed in the tumor panel. In the 490 metaphases analyzed in 21 tumor samples, when the ploidy status of metaphases is taken into consideration (Table 1), 1311 of each of the 21 chromosomes would be expected if no chromosome gain or loss would have happened. We next counted the actual number of chromosomes present in the tumor material (observed number of chromosomes, Table 3). Percentage of numerical chromosome changes was subsequently calculated for each single chromosome. It appeared chromosome gains were less common (in 9 chromosomes), but more profound (up to 23,34%) compared to chromosome losses (in 11 chromosomes, but up to 14,19%, Table 3 and Figure 2). The most commonly gained chromosome in the material was RNO4 (with the frequency of 23.34%, Figure 2) and the most commonly lost chromosome was RNO15 (with the frequency of 14.19%, Figure 2).

Amplification of the proximal region of RNO4 has previously been reported as the most common aberration in BDII rat EC tumors by comparative genome hybridization (CGH) [3,22]. Walentinsson *et al*. [32] further reported the genes *Cdk6 *(cyclin-dependent kinase 6) and *Met *(hepatocyte growth factor receptor) as the main targets for the observed gene amplifications and thus suggested that up-regulation of Cdk6 and/or Met may contribute to the development of endometrial cancers in the BDII rat model. Whether the biological significance of the observed RNO4 gains in the present work is comparable to the earlier reported gene amplifications in this chromosome remains to be investigated.

In earlier studies, losses in the short arm of RNO15 were reported as one of the most characteristic change detected by CGH in BDII rat ECs [3,22]. RNO15 is homologous to segments of human chromosomes 10q, 6p, 3p, 14q, 8p, and 13q, some of which are reported to exhibit loss of heterozygosity and deletions in human endometrial cancers [33-35] and in other human cancer types [36]. There are a number of important cancer-related genes located on this chromosome, including *Anxa7 *(annexin 7, its human counterpart *ANXA7 *located on HSA10q21), which is a tumor suppressor gene associated with prostate cancer [37] and *Bmp4*, *Lgals3 *and *Cdkn3*, whose human counterparts are located on chromosome band 14q22 in human. *BMP4 *(bone morphologic protein 4) was shown to be associated with poorly differentiated gastric cancer and in bone and soft tissue sarcoma [38,39]. Association of *LGALS3 *(lectin, galactoside-binding soluble 3) is reported with endometrial, breast and colorectal cancer [40-42], and *CDKN3 *(cyclin-dependent kinase inhibitor 3) is known to be involved in hepatocarcinogenesis and breast and prostate cancer development [43,44].

We next investigated frequency of non-random structural chromosomal changes in this material. To address this, we recorded structural aberrations (amplification, deletion and translocation) for all of the chromosomes, metaphases and tumor samples (Table 4). Using the Pearsson coefficient of correlation test, we examined whether the total number of observed structural changes in the tumor panel would correlate to the total size of the genome. The analysis revealed that no such correlation existed (r_sTOT _= 0,351, df = 19, P > 0,05), indicating that the observed alterations could not be explained by random events in the genome. Repeating the analysis, this time for the individual chromosomes, we found that the observed lack of correlation was mainly due to nonrandom aberrations in six chromosomes: RNO3, RNO6, RNO10, RNO11, RNO12, and RNO20. For these chromosomes, frequencies of observed changes per chromosome were higher than could be explained by random events corresponding to the genomic content of each of the chromosomes. In five of these chromosomes (RNO3, RNO6, RNO11, RNO12 and RNO20) over 80% of the changes were in form of translocations, whereas deletions and translocations were equally prevalent in the sixth, i.e.RNO10.

**Figure 3:**
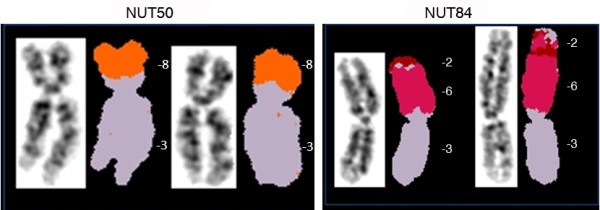
**Examples of chromosomal translocations involving short arm of RNO3 as revealed by SKY analysis**. The translocation breakpoint in RNO3p appeared to be at the similar location in many tumors.

Chromosomal translocations in tumor material can be classified in two major groups: the tumor-specific translocations, i.e. those that occur at specific cytogenetic band in a particular chromosome in several tumor samples and types. The second group is those that occur randomly at different positions of the chromosomes. SKY analysis of 21 BDII rat ECs showed that both groups of translocations were present in the tumor panel (Table 2). Unbalanced chromosomal translocations involving RNO3 were the most commonly observed structural changes in the tumor material. RNO3 translocations seemed to be non-random, since in the majority of cases (8 out of 14, 57%) the breakpoint was in the short arm of RNO3, often at the cytogenetic band RNO3p11 (Figure 3), which harbors ribosomal genes. Recurrent unbalanced translocations of short arm of RNO3 have earlier been reported and discussed in transformed rat mammary epithelial cell lines [45]. Furthermore, loss of RNO3p has been reported in a number of transformed rat cell lines and *in vivo *hepatic lesions [46,47] and are suggested to be involved in mitotic spindle malfunction and thus aneuploidy in these models [48]. Taken together, these data suggest RNO3p may contain one or several genes that fit the tumor suppressor paradigm, as loss of this region has repeatedly reported in a number of tumor models, including the model presented in this report, in mammary tumors as well as in other rat malignancies of epithelial origin [45-47]. RNO3p is homologous to segments of human chromosome bands 2q13 and 2q22 as well as to a larger segment of human chromosome 9, including cytogenetic bands 9q33-q34.

The second most commonly observed chromosomal translocations was in RNO11 (in 11 tumors, 52%). In the majority of cases RNO11 breakpoints were detected in the short arm of the chromosome, where, similar to RNO3p, harbors ribosomal genes.

RNO10 was an interesting chromosome in this analysis, since translocation and/or partial deletion of the distal part of the chromosome was observed in 17 tumors (71%). Nine tumors (NUT6, NUT7, NUT84, NUT100, NUT127, RUT2, RUT6, RUT7 and RUT25) showed only deletion, four (NUT7, NUT127, RUT2 and RUT25) both deletion and translocation and three (NUT3, NUT97 and NUT128) displayed only translocation in distal RNO10. Since deletions of distal part of RNO10 were detected in more than half of the tumors, we propose that the most direct outcome of RNO10 translocations might be loss of an important tumor suppressor activity(ies) with important implications in endometrial carcinogenesis, at least in this tumor model analyzed. Earlier molecular data confirms and extends this theory, as recurrent allelic imbalances/loss of heterozygosity in three independent regions of distal RNO10 have earlier been reported in BDII rat ECs [49-51].

## Conclusions

In conclusion, we found SKY analysis a valuable technique for detailed cytogenetic analysis of experimental tumors. SKY analysis of 21 experimental ECs developed in a well-characterized rat tumor model revealed non-random numerical and structural chromosome changes, including gain of RNO4, loss of RNO15, and structural changes in RNO3, RNO6, RNO10, RNO11, RNO12, and RNO20. The non-random nature of these events, as documented by their high frequencies of incidence, is suggestive for dynamic selection for these changes during BDII EC tumorigenesis and therefore for their potential contribution into development of this malignancy. Detailed molecular analysis of the identified genetic changes in this study and comparative analysis with the findings in human ECs may provide new insights into underlying mechanisims in EC tumorigenesis.

## List of abbreviations

*Anxa7*: annexin 7; BDII: the BDII/Han rat strain; *Bmp4*: bone morphologic protein 4; BN: the BN/Han rat strain; *Cdk6*: cyclin-dependent kinase 6; *Cdkn3*: cyclin-dependent kinase inhibitor 3; CGH: comparative genome hybridization; CIN: chromosome instability; F1: first generation progeny of intercross; F2: second generation progeny of intercross; EC: endometrial carcinoma; *Lgals3*: lectin; galactoside-binding soluble 3; *Met*: hepatocyte growth factor receptor; N1: backcross progeny; NUT: N1 uterine tumor; REF: rat embryo fibroblast; RNO: rat chromosome; RUT: Rat uterine tumor; i.e. tumors developed in the F1 and F2 progeny; SPRD: the SPRD-Cu3/Han rat strain; SKY: spectral karyotyping.

## Competing interests

The authors declare that they have no competing interests.

## Authors' contributions

EF carried out cell chromosome preparations and SKY experiments, performed data analysis and helped to draft the manuscript. CH participated in chromosome preparations and SKY analysis. KKL participated in the analysis of data as well as helped to draft the manuscript. AB conceived of the study, participated in its design and coordination and drafted the manuscript. All authors read and approved the final manuscript.
